# A refined method of quantifying deceleration capacity index for heart rate variability analysis

**DOI:** 10.1186/s12938-018-0618-x

**Published:** 2018-12-18

**Authors:** Hongyun Liu, Ping Zhan, Jinlong Shi, Guojing Wang, Buqing Wang, Weidong Wang

**Affiliations:** 1 Department of Biomedical Engineering, Chinese PLA General Hospital, Room_105, South Ward Building, No. 28 Fuxing Road, Haidian District, Beijing, 100853 China; 2 Center of Medical Device R & D and Clinical Evaluation, Chinese PLA General Hospital, Beijing, 100853 China

**Keywords:** Phase-rectified signal averaging, Deceleration capacity, Autonomic nervous system, Heart rate variability

## Abstract

**Background:**

Phase-rectified signal averaging (PRSA) was often applied to assess the cardiac vagal modulation. Despite its broad use, this method suffers from the confounding effects of anomalous variants of sinus rhythm. This study aimed to improve the original PRSA method in deceleration capacity (DC) quantification.

**Methods:**

The refined deceleration capacity (*DC*_*ref*_) was calculated by excluding from non-vagally mediated abnormal variants of sinus rhythms. Holter recordings from 202 healthy subjects and 51 patients with end-stage renal disease (ESRD) have been used for validity. The *DC*_*ref*_ was compared to original DC (*DC*_*org*_) by the area under receiver operating characteristic curve.

**Results:**

Experimental results demonstrate that the original and refined *DC*s calculated from 24-h, 2-h, and 30-min Holter recordings are significantly lower in patients with ESRD than those in the healthy group. In receiver operating characteristic curve analysis, the *DC*_*ref*_ provides better performance than the *DC*_*org*_ in distinguishing between the patients with ESRD and healthy control subjects. Furthermore, the refined PRSA technique enhances the low frequency and attenuates high frequency components for spectral analysis in ESRD patients.

**Conclusions:**

The *DC*_*ref*_ appears to reduce the influence of non-vagally mediated abnormal variants of sinus rhythm and highlighting the pathological influence. *DC*_*ref*_, especially assessed from short-term electrocardiography recordings, may be complementary to existing autonomic function assessment, risk stratification, and efficacy prediction strategies.

## Introduction

The autonomic nervous system (ANS) plays an essential and important role in maintaining homeostasis for the body’s internal environment via two opposing branches: the sympathetic system and the parasympathetic/vagal system [1, 2]. Autonomic imbalance, characterized by a hyperactive sympathetic system and a hypoactive parasympathetic system, is associated with various pathological conditions [3, 4]. Heart rate variability (HRV), affected by both sympathetic and vagal modulations, is considered a non-invasive manifestation of neurocardiac function that reflects ANS dynamics and heart–brain interactions [2]. Standard time domain, frequency domain and non-linear measures of HRV are emerging as descriptors to provide information on the nervous system organization of homeostatic responses in accordance with the situational requirements, and some metrics are used as dynamical biomarkers of cardiac vagal modulation [5].

Phase-rectified signal averaging (PRSA) was introduced to identify subtle short-term repeated patterns for characterizing the complex non-linear, non-stationary and quasi-periodic signals [6]. When applied to heartbeat time series, the deceleration capacity (DC) of heart rate quantified by PRSA also reflects parasympathetic/vagal control of heart rate [7]. Despite the growing application of DC [8–13], the method is affected by one shortcoming: non-vagally mediated abnormal variants of sinus rhythms in heartbeat interval time series are used to quantify DC, thus confounding the evaluation of cardiac vagal modulation. Previous studies reported that the presence of erratic sinus rhythm, sinus alternans, and their variants, influence values of HRV indices including root-mean-square of successive differences of normal-to-normal interbeat intervals (rMSSD), the percentage of normal-to-normal intervals > 50 ms different from the previous interval (pNN50), and the amount of variance in normal-to-normal intervals at respiratory frequencies (HF, 0.15–0.4 Hz) [14–16]. This distorted beat-to-beat variability, which does not provide a normal assessment of cardiac autonomic function, consequently leading to paradoxical interpretation between HRV indices and cardiac health. Furthermore, DC and traditional HRV measures that are believed to reflect cardiac vagal modulation might have relatively little predictive power for outcomes due to the confounding effects of non-vagally mediated abnormal variants of sinus rhythms.

The purpose of this study is to develop and investigate a refined version of PRSA to quantify DC, represented as refined deceleration capacity (DC_ref_), by excluding from non-vagally mediated abnormal variants of sinus rhythms in heartbeat interval time series to counteract the shortcoming. This approach is expected to be more accurate than original PRSA in assessing cardiac vagal modulation. Experimental data including long-term electrocardiography (ECG) recordings were obtained from healthy and end-stage renal disease (ESRD) subjects. ESRD is characterized by altered cardiac autonomic function, reflecting as vagal modulation damage and sympathetic nerve overactivity [17]. The comparison between original deceleration capacity (*DC*_*org*_) and *DC*_*ref*_ quantified from different ECG-regimens in healthy subjects and patients with ESRD is utilized to demonstrate the performance of the two approaches over real clinical data.

## Materials and methods

### The original PRSA method

Traditional analyses used to analyze signals that are recorded from composite systems over a prolonged period of time are usually spectral analysis and cross-correlation analysis. However, non-stationary and non-linear are major problems of both methods [6]. When applied to HRV signal, the PRSA technique reduce the signal to a much shorter sequence keeping all relevant quasi-periodicities but eliminating non-stationarities, artifacts, and noise. This offering the possibility to separate characterizations of deceleration-related and acceleration-related modulations which might provide more differentiated insights into cardiac autonomic regulation processes [6, 7]. DC, quantified by PRSA with the solely deceleration-related process in heartbeat interval time series, is a measure of cardiac vagal modulation [7]. The DC quantification for HRV signal is essentially based on four steps: (1) definition of anchor points according to certain properties of the heartbeat interval time series; (2) definition of surroundings with certain length around each anchor point; (3) the PRSA signal is obtained by averaging the signals within the segments which are aligned at the anchors; and (4) DC is quantified corresponding to a computation of PRSA signal by Haar wavelet analysis [6, 7].

#### Definition of deceleration-related anchors

DC is computed based on heartbeat interval time series $RR = \{ RR_{i} \}$ derived from ECG recordings. In the simple version of the PRSA algorithm, for computation of DC, heartbeat intervals longer than the preceding interval are identified as deceleration-related anchors ($RR_{{i_{\gamma } }} , \, \gamma = 1, \ldots ,M$, M is the total number of anchor points).

1 $$RR_{i} > RR_{i - 1}$$

Heartbeat interval time series is almost always interfered by technical and physiological artifacts due to the computational algorithm, ectopic beats and arrhythmic events which must not be qualified as anchor points. To overcome this problem, heartbeat interval prolongations of more than a certain threshold are excluded. However, heartbeat intervals with high-frequency fluctuations that compete with or even exceed the shortest-term modulatory responsiveness of the vagal system still enters the DC quantification process, thus distort high-frequency variability not due to cardiac vagal modulation.

#### Definition of segments

Segments of heartbeat interval time series with a length of *2L* are defined around each anchor point $RR_{{i_{\gamma } }} , \, \gamma = 1, \ldots ,M$. Anchor points close to the beginning or the end of the time series, where no full surroundings of length *2L* are available, are disregarded. Segments that surround adjacent anchors can overlap. The heartbeat intervals in segment number $\gamma$, corresponding to anchor point $RR_{{i_{\gamma } }}$ are as following.

2 $$RR_{{i_{\gamma } - L}} , \, RR_{{i_{\gamma } - L + 1}} , \ldots , { }RR_{{i_{\gamma } }} , \ldots , { }RR_{{i_{\gamma } + L - 2}} , \, RR_{{i_{\gamma } + L - 1}}$$

#### Phase rectification and signal averaging

All segments $\gamma , \, \gamma = 1, \ldots ,M$ are aligned and centered at their anchor points $RR_{{i_{\gamma } }}$ and the PRSA signal $\overline{RR} (k)$ is obtained by averaging over all segments.

3 $$\overline{RR} (k) = \frac{1}{M}\sum\limits_{\gamma = 1}^{M} {RR_{{i_{\gamma } + k}} \, } k = - L, - L + 1, \ldots ,0, \ldots ,L - 2,L - 1$$

In this average, non-periodic components cancel out, and only events that have a fixed phase relationship with the anchor points, i.e., all periodicities and quasi-periodicities, ‘survive’ the procedure. $\overline{RR} (k)$ represents important features of the original heartbeat interval data containing all quasi-periodicities aligned with phase zero in the center (at k = 0).

#### Quantification of DC

PRSA signal $\overline{RR} (k)$ is quantified by Haar wavelet analysis, where the scale of 2 is used to calculate DC.

4 $$DC_{org} { = }[\overline{RR} (0) + \overline{RR} (1) - \overline{RR} ( - 1) - \overline{RR} ( - 2)]/4$$

$\overline{RR} (0)$ is defined as the average of the heartbeat intervals at all anchor points, $\overline{RR} (1)$ and $\overline{RR} ( - 1)$ are the averages of the heartbeat intervals immediately following and preceding the anchor points, etc.

### Refined DC quantification

Evidence suggests that erratic rhythm can be a consequence of high sympathetic activation [18]. It is also speculated that the observed non-vagally mediated HRV also termed as heart rate fragmentation including erratic sinus rhythm, sinus alternans, and their variants is originated from the breakdown of one or more components of the regulatory network controlling heart rate dynamics [18–20]. Moreover, this fragmentation or anomalous short-term HRV are marked by abrupt changes in the sign of heart rate deceleration/acceleration can be quantified by statistical analysis of inflection points or zero-crossing points [19]. To address this confounding effects of non-vagally mediated HRV, which may distort DC not due to cardiac vagal modulation, we proposed a refined PRSA technique to quantify DC by excluding inflection points in heartbeat interval time series.

#### Definition of inflection points

**Fig. 1:**
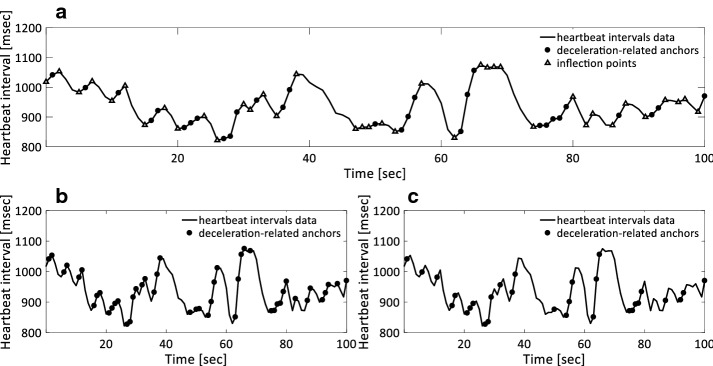
Illustration of anchor points selection. **a** Anchor points for a real ECG recording. Deceleration-related anchor points selected from heartbeat interval time series according to original PRSA (**b**) and modified PRSA (**c**) method, respectively

For given ECG recordings, the time series of heartbeat intervals, $\{ RR_{i} \} = \{ t_{{R_{i} }} - t_{{R_{i - 1} }} \}$, where $t_{{R_{i} }}$ represents the time of occurrence of the *i*th sinus beat, and the time series of the differences between consecutive heartbeat intervals (increments), $\{ \Delta RR_{i} \} = \{ RR_{i} - RR_{i - 1} \}$, were derived. The inflection points in the heartbeat interval time series, or equivalently, the zero-crossing points in the increment time series are defined as shown in Fig. 1.

A $t_{{R_{i} }}$ represents an inflection point if Eq. (5) is satisfied, that is, if $t_{{R_{i} }}$ is an instant of inversion of heart rate deceleration/acceleration sign or of change to or from zero.

5 $$\Delta RR_{i} \times \Delta RR_{i + 1} \le 0$$

#### Quantification of refined DC

After the identification of deceleration-related anchor points and inflection points are selected according to Eqs. (1) and (5), respectively (Fig. 1). Segments with the length of *2L* are identified around each deceleration-related anchor. The refined PRSA signal is obtained by averaging over all segments, which are aligned and centered at deceleration-related anchors. The refined DC (*DC*_*ref*_) is quantified according to Eq. (5). Consequently, omitting the segments centered at inflection points will reduce or eliminate the effects of heart rate fragmentation and improve the performance of the PRSA technique to assess the cardiac vagal tone modulation.

### Experimental data and analysis procedure

We employed two long-term (24-h) ECG ambulatory databases which are made available via the University of Rochester Telemetric and Holter ECG Warehouse (THEW) archives (http://thew-project.org/databases.htm). In addition to ECG data, automated beat annotations are available after reviewing and adjudicating manually in both databases. All human data was obtained retrospectively from completed, Institutional-Review-Board-approved clinical research studies with subject de-identification. These trials complied with the Declaration of Helsinki and all subjects signed informed consent documents.

The Healthy database (THEW identification: E-HOL-03-0202-003) comprises 24-h Holter recordings from 202 ostensibly healthy subjects (102 males). Subjects had (1) no overt cardiovascular disease or history of cardiovascular disorders; (2) no reported medications, (3) a normal physical examination, (4) a 12-lead ECG showing sinus rhythm with normal waveforms (or a normal echocardiogram and normal ECG exercise testing in the presence of any questionable findings ECG changes). The ECG signals were recorded at a sampling frequency of 200 Hz. Overall, we analyzed data from 191 healthy adult subjects by excluding from 11 subjects with poor ECG quality. The ESRD database (THEW identification: E-HOL-03-0202-016) comprises 48-h Holter recordings with a sampling frequency of 1000 Hz from 51 ESRD patients with high risk for cardiac arrhythmias and sudden cardiac death (30 males). ESRD subjects confirmed the history of hypertension or diabetes requiring treatment enrolled in the study. Exclusion criteria included a history of chronic atrial fibrillation, with class I antiarrhythmic, pacemaker, implantable cardioverter defibrillator device, cardiac resynchronization therapy device, female subject of childbearing potential not using medically prescribed contraceptive measures and subject unable to cooperate with the protocol due to dementia, psychological, or other related reason.

For our analysis, all 24-h Holter recordings were analyzed automatically and annotated files were then carefully inspected and corrected by technicians for extracting the RR intervals. Furthermore, any RR-interval that exhibited more than 20% change from the previous RR-interval were excluded, as they were likely to be related to premature beats, artifacts and measurement noise [21]. 2-h and 30-min episodes of heartbeat intervals without exercise and naps within daytime (between 8 a.m. and 5 p.m.) were extracted from each recording for PRSA analysis. All ECG segments were selected from the same period to reduce confounding effects of the circadian rhythm and physical activity. Measures of DC for long-term (24-h), 2-h and 30-min ECG recordings were calculated based on the original and the proposed PRSA method, respectively.

### Statistical analysis

Data were presented as the mean ± standard deviation (SD) for continuous variables. Gaussian distribution and homogeneity of variance tests were applied to determine the distribution and homoscedasticity of sample data. Because of the non-normal distribution and heterogeneity of variance of some sample data, a Mann–Whitney U test was applied to compare DCs between groups (healthy vs. ESRD). Correlation analysis was perfomed between *DC*_*org*_ and *DC*_*ref*_ using Spearman’s correlation test. In order to compare the ability of *DC*_*org*_ indices and *DC*_*ref*_ to differentiate the ESRD patients from the healthy control subjects, receiver operating characteristic curve (ROC) was constructed from the sensitivity and specificity after leave-one-out cross-validation of the continuous variables to assess the goodness of fit of each model. The area under the ROC curve (AUCs) gave an estimate of the overall discriminate ability. All statistical analyses were performed using SPSS version 20 software package (SPSS, Chicago, Ill, USA). All the p values were adjusted using the false discovery rate (FDR) method and a value of p < 0.05 was considered to indicate statistical significance.

## Results

**Fig. 2:**
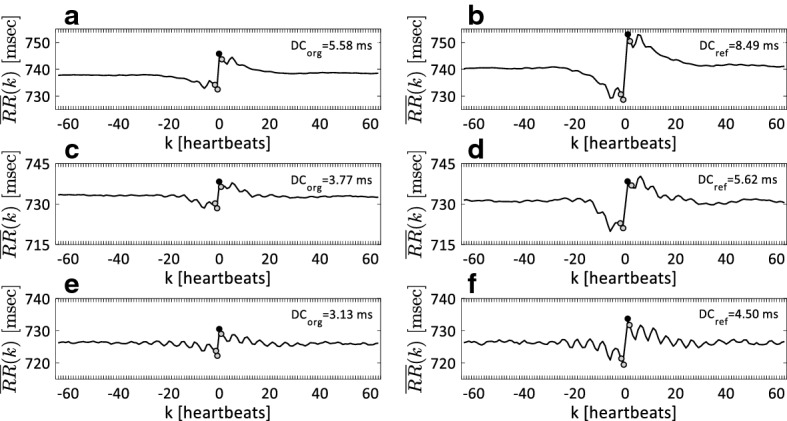
Representative original and refined PRSA signals of 24-h (**a**, **b**), 2-h (**c**, **d**) and 30-min (**e**, **f**) recordings of heartbeat intervals in one patient with end-stage renal disease (ESRD)

Demographic data and clinical factors of ESRD patients and healthy control subjects are presented in Table 1. Figure 2 shows the deceleration-related PRSA signal and DCs of 24-h, 2-h and 30-min recordings of heartbeat intervals in a patient with ESRD. The segment length *L* for both the original and refined PRSA methods is set to 64. Similar patterns of the original and refined PRSA curves were observed for both long-term (24-h) and short-term (2-h and 30-min) ECG recordings. The heartbeat interval oscillations in the refined PRSA curves extracted from different length of ECG recordings are enhanced, especially around the deceleration-related anchor points. Different levels of the heartbeat intervals obtained by the original and refined PRSA methods can also be observed. As the length of the analyzed ECG signal is shortened, the difference between original and refined PRSA signals is also reduced.

**Table 1:** Demographic data and clinical factors of all study population

Variables	Healthy (n = 191)	ESRD (n = 51)
Male/female	96/95	30/21
Age (year)	38.0 ± 15.5	60.0 ± 11.9
Height (cm)	168.8 ± 10.4	NA
Weight (kg)	69.6 ± 15.2	NA
BMI (kg/cm^2^)	20.07 ± 4.44	NA
BP_SYS (mmHg)	116 ± 13	139 ± 22
BP_DIA (mmHg)	75 ± 8	76 ± 13
Diabetes Mellitus, n (%)	NA	32 (62.7%)
Hypertension, n (%)	NA	48 (94.1%)
LVEF (%)	NA	59 ± 16
Glucose (mg/dL)	NA	121 ± 49
Calcium (mg/dL)	NA	8.9 ± 1.6
Phosphate (mg/dL)	NA	5.2 ± 1.4
Albumin (g/dL)	NA	4.0 ± 0.4
Hemoglobin (g/dL)	NA	13.1 ± 12.3
Creatinine (mg/dL)	NA	8.6 ± 2.8

*NA* not available, *BMI* body mass index, *BP_SYS* systolic blood pressure, *BP_DIA* diastolic blood pressure, *LVEF* left ventricular ejection fraction, *ESRD* end-stage renal disease

**Fig. 3:**
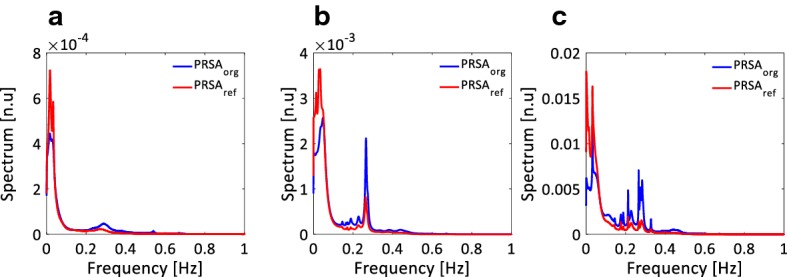
The averages of the normalized spectra obtained with the original and refined PRSA signal in the end-stage renal disease (ESRD) group for 24-h (**a**), 2-h (**b**), and 30-min (**c**) recordings of heartbeat intervals

The averages of the normalized spectra obtained with the original and refined PRSA signal in the group of 51 patients with ESRD are depicted in Fig. 3. Refined PRSA method clearly enhances the power of low frequency (LF, 0.04–0.15 Hz) and attenuates the power of high frequency (HF, 0.15–0.4 Hz) components for 24-h, 2-h and 30-min ECG recordings, respectively.

DCs calculated based on original and refined PRSA methods with different criteria of anchor point selection for the healthy control subjects and the ESRD group are given in Table 2. For the healthy group, we compared DCs quantified by original and modified PRSA methods. *DC*_*ref*_ values were significantly higher than *DC*_*org*_ for all 24-h (mean difference 4.32 ± 1.68, p < 0.001), 2-h (mean difference 4.64 ± 1.97, p < 0.001)and 30-min (mean difference 4.72 ± 2.03, p < 0.001) ECG segments. Furthermore, the Pearson’s correlation coefficients between the *DC*_*org*_ and *DC*_*ref*_ for 24-h, 2-h and 30-min ECG recordings were 0.980, 0.983 and 0.981 (all p < 0.001), respectively.

**Table 2:** Measures of original and refined deceleration capacity in healthy and ESRD groups

Variables	Healthy (n = 191)	ESRD (n = 51)	p value	AUC	Youden’s index
24-h					
*DC*_*org*_ (msec)	13.43 ± 5.60	4.36 ± 2.60	< 0.001	0.945 ± 0.017	0.766
*DC*_*ref*_ (msec)	17.77 ± 6.79	6.25 ± 3.59	< 0.001	0.944 ± 0.018	0.774
2-h					
*DC*_*org*_ (msec)	11.94 ± 5.28	3.50 ± 1.87	< 0.001	0.960 ± 0.013	0.802
*DC*_*ref*_ (msec)	16.61 ± 6.94	4.81 ± 2.39	< 0.001	0.969 ± 0.011	0.842
30-min					
*DC*_*org*_ (msec)	12.40 ± 5.46	3.25 ± 1.94	< 0.001	0.967 ± 0.012	0.825
*DC*_*ref*_ (msec)	17.14 ± 7.12	4.53 ± 2.60	< 0.001	0.971 ± 0.011	0.830

*DC*_*org*_ original deceleration capacity, *DC*_*ref*_ refined deceleration capacity, *ESRD* end-stage renal disease, *AUC* area under receiver operating characteristic curve

**Fig. 4:**
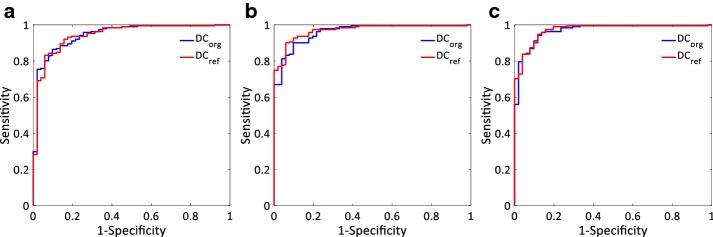
Analysis of the discrimination power of the healthy and end-stage renal disease (ESRD) groups by receiver operating characteristic curve (ROC) analysis. The areas under the curve (AUC) of *DC*_*org*_ and *DC*_*ref*_ were 0.945 vs. 0.944, 0.960 vs. 0.969, and 0.967 vs. 0.971 for **a** 24-h, **b** 2-h and **c** 30-min ECG recordings, respectively

ESRD patients substantially had significantly lower *DC*_*org*_ and *DC*_*ref*_ compared to healthy control subjects for the analyzed three lengths of ECG episodes (all p < 0.001). In ROC curve analysis, The areas under the curve (AUC) of *DC*_*org*_ and *DC*_*ref*_ were 0.945 vs. 0.944, 0.960 vs. 0.969, and 0.967 vs. 0.971 for 24-h, 2-h and 30-min ECG recordings, respectively (Table 2 and Fig. 4). The AUC of *DC*_*ref*_ from long-term (24-h) ECG recordings was comparable to that of *DC*_*org*_ while the AUCs of *DC*_*ref*_ from short-term (2-h and 30-min) ECG recordings were slightly higher than those of *DC*_*org*_. In addition, the maximum of Youden’s index of long-term (24-h) *DC*_*ref*_ was higher than that of short-term (2-h and 30-min) *DC*_*org*_. *DC*_*ref*_ from 2-h ECG recordings, providing the highest value of Youden’s index, had the best discrimination power of all calculated DCs. The best cutoff value of *DC*_*ref*_ from 2-h ECG episodes for distinguishing ESRD from healthy subjects was 8.26 ms, with a sensitivity of 90.1%, specificity of 94.1%.

## Discussion

In the present study, a refined method was presented for the quantification of deceleration capacity of heart rate. *DCs* were calculated through proposed refined and original PRSA methods from different length of ECG recordings and were tested their predictive value in healthy subjects and patients with ESRD. The present findings demonstrate that the refined PRSA technique better reflects the periodic components in heartbeat intervals and enhances the HF and LF components for spectral analysis. *DC*_*ref*_, with significantly higher values compared to *DC*_*org*_, was significantly lower for ESRD group than that with healthy group. This phenomenon was observed for the analysis of 24-h, as well as 2-h and 30-min ECG recordings. Furthermore, the *DC*_*ref*_ quantified from different length of ECG episodes outperformed corresponding *DC*_*org*_ in separating healthy subjects from patients with ESRD. Among them, *DC*_*ref*_ obtained through 2-h ECG recordings have the best discrimination ability.

HRV analysis is a non-invasive, simple and effective method for assessing the sympathovagal balance of ANS and is used to predict the risk to many cardiovascular diseases and neurological disorders [4, 5, 22, 23]. The analysis of blood pressure variability (BPV) has also been used to evaluate cardiovascular autonomic regulatory function [24, 25]. However, conventional linear time and frequency analyses are often applied to calculate measures of HRV and BPV, even though the regulation of the ANS on cardiac activity is considered to be a nonlinear physiological activity [26, 27]. Despite the widespread application of HRV, linear approaches to HRV signals may introduce intrinsic computational errors [28–30]. Moreover, since traditional linear HRV analyses cannot accurately distinguish the vagal and sympathetic activities of the ANS, the interpretation of the HRV measures is more complex than generally appreciated, and there is potential for incorrect conclusions and for excessive or unfounded extrapolations [5, 21, 23, 27]. Therefore, efficient methods for characterizing the complex non-linear, non-stationary and quasi-periodic ECG signal remain to be established. In 2006, PRSA method has been introduced to make possible an approximate distinction of autonomic control effects on sinus node, by selectively assessing the capacity of heart rate to decelerate and accelerate, representing an indirect integrated quantification of the activities of the autonomic systems. Since the PRSA technique is able to robustly extract and enhance periodic quasi-periodic oscillations masked by noise and artifacts from complex signals, it has been proven to yield stronger prognostic power than the traditional parameters of HRV among post-myocardial infarction patients [7]. The main characteristic of *DC*_*ref*_ in the present study is the use of stricter criteria for the selection of the deceleration-related anchor points. This allows a smaller number of legal vagally-mediated anchor points to participate in the quantification, thereby improving the accuracy of cardiac vagal modulation assessment.

Theoretically, DC index could reflect the cardiac vagal modulation on heart rate [7, 10, 13, 31, 32]. When applied PRSA to heartbeat intervals for assessing vagal tone modulation, the anchor points selected according to Eq. (1) are expected to be vagally mediated and change in a progressive way. However, non-vagally mediated HRV with more frequent changes in heart rate deceleration/acceleration sign heart rate changes has a distinct dynamical signature, namely. In the “extreme” case of sinus alternans, the sign of heart rate acceleration changes every beat [19]. The presence of these abnormal variants of sinus rhythm limits the utility of original PRSA for HRV analysis since an increase in the overall amount of short-term variability can no longer be solely attributed to enhanced vagal tone modulation. Pan et al. applied sinusoidal signal analysis to anchor point selection to improve the PRSA processing, and proved that the modified DC is superior to the conventional approach in distinguishing chronic heart failure cases [33]. Sacha et al. showed that the lower anchors filtering threshold could decrease the correlation between DC and high-frequency fluctuations [34]. However, the influences of anchors filtering with different thresholds on the prognostic or discriminative power remain to be elucidated. Piskorski et al. defined heart rate asymmetry by acceleration and deceleration runs, which was reduced in post-infarction patients with increased risk of mortality [35]. Arsenos et al. also proposed DC_sgn_ and BBDC, which can discriminate more efficiently healthy young and elderly subjects than the original PRSA method [36]. However, the interpretation of the features of DC and AC were not linked to specific physiological regulatory mechanisms in the process of improving the PRSA technique. Nasario-Junior et al. introduced a DC adaptation from the perspective of synchronization and improved the AUC diagnostic accuracy of DC to assess physical conditioning [37]. According to the physiological origins and mechanisms of HRV, the present study proposed a refined quantitative approach, which excluding from non-vagally mediated inflection points to quantify DC for assessing the cardiac vagal tone modulation. The results of our study showed that the refined PRSA method could enhance the power of LF and attenuate the power of LF components compared with original PRSA technique. This is consistent with our understanding of the refined PRSA method, which allows higher accuracy for detecting the most important frequencies, especially the HF components by addressing the confounding effects of heart rate fragmentation. Since HF is usually interpreted as a measure of cardiac vagal tone modulation, the refined PRSA may provide a promising method for accurate assessment of cardiac vagal tone modulation and integrate probe of the cardiac neuroautonomic-electrophysiologic regulatory system.

It has been observed that an imbalance between cardiac vagal and sympathetic modulation occurs and that a sympathetic activation is an early event in the pathophysiology of ESRD [38, 39]. In our study, the significant lower DC values suggest that patients with ESRD were in a lower vagal modulating state compared with healthy subjects. Since there exist reciprocal changes in the interaction of sympathetic and parasympathetic nervous systems, that is the activation of sympathetic nerve is accompanied by the withdrawal of parasympathetic nerve, and vice versa. The sympathetic activation in ESRD might be a secondary phenomenon. Vagal denervation, which weakened antagonism to sympathetic activity, is most likely to induce the abnormal activation of the sympathetic nerve. The phenomenon that enhanced LF band and attenuated HF band by the proposed refined PRSA technique is consistent with our understanding of antagonistic mechanisms of the sympathetic and parasympathetic nervous system. Furthermore, the proposed *DC*_*ref*_ in the present study was able to improve the classification of healthy subjects from ESRD patients compared to *DC*_*org*_. In addition to showing the higher AUC values (except for 24-h ECG recordings) among all methods tested, the *DC*_*ref*_ also showed higher Youden’s index, thus featuring better discrimination power. Most importantly, *DC*_*ref*_ from 2-h ECG recordings had the best discrimination power of all calculated DCs. Similar with a recent study [40], our findings also indicate that short-term *DC*_*ref*_, especially from 2-h ECG recordings may be a more useful and better index for autonomic bedside risk stratification that as complementary to traditional HRV measures and clinical factors.

Several limitations are present in this study. First, the sample size is small for ESRD group. The ESRD patients enrolled after strictly screening through enrollment exclusion and criteria was most likely to be in a similar autonomic dysfunction, leading to the advantage of *DC*_*ref*_ is not particularly obvious. Second, only three ECG-regimes, the 24-h, 2-h and 30-min ECG recordings were used to quantify DCs. More ECG-regimens should be used to determine the optimal ECG length for *DC*_*ref*_ quantification, which could be performed in everyday clinical practice. Third, the proposed *DC*_*ref*_ was verified by separating healthy subjects from ESRD patients. Since the original PRSA method was developed as a prognostic tool for risk stratification, particularly mortality in post-myocardial infarction patients. Further studies are warranted to demonstrate advantages of *DC*_*ref*_ in autonomic risk stratification.

## Conclusions

The refined deceleration capacity index (*DC*_*ref*_), which excludes from non-vagally mediated inflection points, provides an accurate assessment of cardiac vagal modulation. Furthermore, *DC*_*ref*_ also provides a better distinction between healthy subjects and patients with ESRD compared with *DC*_*org*_. *DC*_*ref*_ derived from 2-h ECG recordings, which have the best discrimination ability, might be complementary to existing autonomic function assessment, risk stratification, and efficacy prediction techniques for clinical use.

## Abbreviations

PRSA: phase-rectified signal averaging
DC: deceleration capacity
ESRD: end-stage renal disease
ANS: autonomic nervous system
HRV: heart rate variability
rMSSD: root-mean-square of successive differences of normal-to-normal interbeat intervals
pNN50: the percentage of normal-to-normal intervals > 50 ms different from the previous interval
ECG: electrocardiography
ROC: receiver operating characteristic curve
AUC: area under ROC curve
LF: low frequency (0.04–0.15 Hz)
HF: high frequency (0.15–0.4 Hz)
